# F163. STRUCTURAL AND FUNCTIONAL ALTERATIONS IN THE BRAIN DURING WORKING MEMORY IN MEDICATION-NAïVE PATIENTS AT CLINICAL HIGH-RISK FOR PSYCHOSIS

**DOI:** 10.1093/schbul/sby017.694

**Published:** 2018-04-01

**Authors:** Kolbjørn Brønnick, Jens Gisselgård, Inge Joa, Aase Ingvild, Robert Jørgensen, Jan Olav Johannessen

**Affiliations:** 1 TIPS – Centre for Clinical Research in Psychosis, Stavanger University Hospital; 2 Stavanger University Hospital

## Abstract

**Background:**

The pre-TIPS study in 1994–95 showed that the duration of untreated psychosis (DUP) was long in our region with a mean value of 2.1 years, and median 26 weeks. This set the stage for the TIPS-study (1997–2000), reducing DUP through information campaigns targeted to the general population and other referral agents (GP’s, schools and others) in Rogaland County (Norway). The information campaigns were launched together with a low threshold organization with direct access to an early detection team, for a diagnostic interview and help. The hypothesis was that this could change help seeking behavior, awareness towards psychosis and thus reduce the DUP. The information campaigns and the early detection team were introduced in an early detection(ED) area (Rogaland county, Norway) comparing DUP with two usual-detection control sites in Oslo (Norway) and Roskilde (Denmark). As a result, DUP in the early detection area was reduced from 26 weeks median to 4 weeks median. Symptom and function advantages of early detection and DUP reduction have been demonstrated as being significant throughout the follow-up period. Social and functional outcome have been increasingly emphasized as being key parameters, as these contribute to both quality of life and to financial costs in society. The TIPS program continues to include and follow patients; since the mid 2000’s also young people with ultra-high risk states (Prevention of Psychosis; POP) or substance induced psychoses.

Previous ultra-high risk studies suggest that psychotic disorders are associated with structural and functional abnormalities within the frontoparietal circuits and that medication status is a potential confounding factor. We investigated neural correlates to ultra-high risk (UHR) for psychosis in medication-naïve patients.

**Methods:**

41 CHR patients and 37 healthy controls were examined with 1.5 Tesla MRI, yielding functional scans while performing an N-back task and structural T1-weighted brain images. Functional and structural data underwent automated preprocessing steps in SPM and Freesurfer, correspondingly. The groups were compared employing mass-univariate strategy within the generalized linear modelling framework.

**Results:**

UHR demonstrated reduced suppression of the medial temporal lobe (MTL) regions during n-back task. We also found that, consistent with previous findings, UHR subjects demonstrated thinning in prefrontal, cingulate, insular and inferior temporal areas, as well as reduced hippocampal volumes.

**Discussion:**

The present findings add to the growing evidence of specific structural and functional abnormalities in the brain as potential neuroimaging markers of psychosis vulnerability.

